# Identification of STAT5A and STAT5B Target Genes in Human T Cells

**DOI:** 10.1371/journal.pone.0086790

**Published:** 2014-01-30

**Authors:** Takahiro Kanai, Scott Seki, Jennifer A. Jenks, Arunima Kohli, Trupti Kawli, Dorrelyn Patacsil Martin, Michael Snyder, Rosa Bacchetta, Kari C. Nadeau

**Affiliations:** 1 Division of Immunology and Allergy, Department of Pediatrics, School of Medicine, Stanford University, Stanford, California, United States of America; 2 Department of Genetics, School of Medicine, Stanford University, Stanford, California, United States of America; 3 San Raffaele Telethon Institute for Gene Therapy (HSR-TIGET), San Raffaele Scientific Institute, Milan, Italy; Emory University, United States of America

## Abstract

Signal transducer and activator of transcription (STAT) comprises a family of universal transcription factors that help cells sense and respond to environmental signals. STAT5 refers to two highly related proteins, STAT5A and STAT5B, with critical function: their complete deficiency is lethal in mice; in humans, STAT5B deficiency alone leads to endocrine and immunological problems, while STAT5A deficiency has not been reported. STAT5A and STAT5B show peptide sequence similarities greater than 90%, but subtle structural differences suggest possible non-redundant roles in gene regulation. However, these roles remain unclear in humans. We applied chromatin immunoprecipitation followed by DNA sequencing using human CD4^+^ T cells to detect candidate genes regulated by STAT5A and/or STAT5B, and quantitative-PCR in *STAT5A* or *STAT5B* knock-down (KD) human CD4^+^ T cells to validate the findings. Our data show STAT5A and STAT5B play redundant roles in cell proliferation and apoptosis via *SGK1* interaction. Interestingly, we found a novel, unique role for STAT5A in binding to genes involved in neural development and function (*NDRG1*, *DNAJC6*, and *SSH2*), while STAT5B appears to play a distinct role in T cell development and function via *DOCK8*, *SNX9*, *FOXP3* and *IL2RA* binding. Our results also suggest that one or more co-activators for STAT5A and/or STAT5B may play important roles in establishing different binding abilities and gene regulation behaviors. The new identification of these genes regulated by STAT5A and/or STAT5B has major implications for understanding the pathophysiology of cancer progression, neural disorders, and immune abnormalities.

## Introduction

Signal transducer and activator of transcription (STAT) comprises a family of universal transcription factors, playing important roles in regulating gene expression in multiple cell types. STAT1 through 6 are essential for transduction of extracellular signals into the cells. STAT5, in particular, plays critical roles in the cellular response to various cytokines and hormones and therefore is crucial to regulation of immune and nervous system functions, as well as cell proliferation and growth, in both humans and rodents [1], [2]. Following cytokine stimulation, the STAT5 protein is rapidly tyrosine phosphorylated, allowing dimerization and translocation to the nucleus, where it binds regulatory regions of target genes [3].

STAT5 encompasses two highly related proteins, STAT5A and STAT5B in humans (Stat5a and Stat5b in rodents). STAT5A and STAT5B show peptide sequence similarities of more than 90%, differing only by 6 amino acids in their DNA binding domains, 20 amino acids in their C-termini [4], and 18 amino acids in their N-termini [5]. These structural differences may result in non-redundant roles for each protein, resulting in unique gene regulation profiles [4], [6]; however this has yet to be clarified in humans.

Previous studies in mice have demonstrated both redundant and non-redundant roles for Stat5a and Stat5b in immune regulation and development. Both Stat5a and Stat5b were essential for normal lymphoid development, and function as critical signal mediators for CD8^+^ T cell homeostasis [7], [8]. Deficiency of only Stat5a resulted in impaired prolactin-dependent mammary cell differentiation [9], whereas deficiency of Stat5b alone resulted in impaired growth [10]. At the same time, human studies suggest differences between human and mouse STAT5-mediated gene regulation that must be taken into consideration. In humans, both male and female patients carrying mutated STAT5B, but with normal levels of STAT5A, have similar growth defects (i.e., there is no sexual dimorphism of body growth rates as has been observed in *Stat5b^−/−^* mice). Moreover, deficiency in both Stat5a and Stat5b murine proteins is required to generate the growth defect observed in human *STAT5B^−/−^* patients [11]. In addition, another study demonstrated different binding abilities for human *IL4RA* between STAT5A and STAT5B, with chromatin immunoprecipitation (ChIP) followed by sequencing (ChIP-seq), although no such difference was observed between Stat5a and Stat5b in mice [12]. Therefore, the data collected thus far on human *STAT5B^−/−^* do not completely recapitulate the immune data reported in *Stat5b^−/−^* mouse models, and suggest there are unique roles for STAT5A and STAT5B in human immune modulation. It is therefore crucial to analyze human samples to elucidate the redundant and non-redundant roles of STAT5A and STAT5B in human gene regulation [13].

We reported that STAT5B deficient patients show severe growth hormone-resistant growth failure despite the presence of normal growth hormone receptor [14], reduced number of natural killer cells and T cells [14], [15], impairment of IL-2 signaling, and decreased regulatory T cell (Treg) number [11]; all these features exist in the presence of normal *STAT5A* expression. Additionally, we have reported that in humans, the anti-apoptotic factor *BCL2L1* is specifically regulated by STAT5A, whereas *FOXP3* and *IL-2RA* expression are specifically regulated by STAT5B [16].

To identify STAT5A and STAT5B target genes, we performed genome-wide ChIP-seq in human CD4^+^ T cells, which are known to express STAT5 upon activation and can be easily expanded [17]. Genes detected by STAT5A and/or STAT5B ChIP-seq were further validated via quantitative RT-PCR (QT-PCR) using siRNA-mediated *STAT5A* or *STAT5B* KD human CD4^+^ T cells.

To the best of our knowledge, this is the first report to show redundant and non-redundant roles of STAT5A and STAT5B in gene regulation in human CD4^+^ T cells.

## Methods

This study was approved by the Stanford Administrative Panel on Human Subjects in Medical Research Institutional Review Board.

### Samples and Cell Isolation

Whole blood samples from healthy adults were obtained from the Stanford Blood Center. CD4^+^ T cells from healthy adults were isolated using RosetteSep® Human CD4^+^ T Cell Enrichment Cocktail (StemCell Technologies, Canada) according to the manufacturer's instructions. The purity of the sorted cells, as assessed by flow cytometry, was found to be greater than 92%.

### Cell culture

Cells were cultured in RPMI 1640 medium (Gibco®, USA) supplemented with 10% fetal bovine serum and 100 µL/mL of antimicrobial agent (Antibiotic-Antimycotic, Gibco®, USA) with PHA-P (5 µg/mL) in a 75 mL flask, and incubated at 37°C in a humidified 5% CO_2_ atmosphere for 3 days. Recombinant human IL-2 (rhIL-2, 100 U/mL) was then added to cultures for either 30 min or 3 days.

### Immunofluorescence staining

CD4^+^ T cells were fixed on a glass slide with 100 µL cold Phosflow Fix Buffer 1 (Becton Dickinson, USA). The cells were permeabilized with 100 µL of Perm/Wash Buffer (Becton Dickinson), blocked with 10% normal donkey serum and 0.3 M glycine in phosphate buffer saline with 0.1% Tween 20, and incubated with anti-STAT5A Ab (sc-1081, Santa Cruz Biotechnology, USA) and anti-STAT5B Ab (135300, Invitrogen, USA) at a dilution of 1∶1,000. Alexa Fluor® 488 donkey anti-rabbit IgG (Invitrogen) or Alexa Fluor® 594 donkey anti-mouse IgG (Invitrogen) was used at a dilution of 1∶1,000 as secondary Ab for STAT5A or STAT5B respectively. The specificities of each Ab were shown in **Fig. S1**. Hoechst 33342, trihydrochloride, trihydrate (Invitrogen) was used at dilution of 1∶10,000 for the nuclear stain.

### Immunoblotting and immunoprecipitation

Cytoplasmic or nuclear proteins from CD4^+^ T cells were extracted using NE-PER Nuclear and Cytoplasmic Extraction Reagents (Thermo Scientific, USA). Lysates were run on a 10% polyacrylamide gel (Bio-Rad, USA) for 30 min at 200 V, transferred to a nitrocellulose membrane (Bio-Rad) for 1 h at 100 V, blocked in 5% milk for 30 min. Proteins were detected by a 1∶1,000 dilution of anti-STAT5A Ab, anti-STAT5B Ab or anti-Phospho STAT5 Ab (Cell Signaling Technology, USA) administered for 18 hours, followed by incubation with anti-rabbit or mouse IgG, HRP-linked Ab (Cell Signaling Technology) at 1∶5,000 for 30 min at room temperature. An enhanced chemiluminescent substrate for detection of HRP (Thermo Scientific) was used for visualization.

Nuclear proteins separated from CD4^+^ T cells were also used for immunoprecipitation using anti-STAT5A Ab or anti-STAT5B Ab with the Protein A/G PLUS-Agarose (Santa Cruz Biotechnology, USA) according to manufacturer's instructions.

### ChIP-seq

CD4^+^ T cells (2.0×10^7^ cells) stimulated by PHA-P for 3 days followed by incubation with rhIL-2 for either 30 min or 3 days were cross-linked with formaldehyde (final concentration, 1%) for 10 min. The reaction was quenched with glycine and cell lysates were sonicated (12 rounds of 20 sec, Sonifier® S-250A, Branson Ultrasonics) and immunoprecipitated with anti-STAT5A Ab (12 µg), anti-STAT5B Ab (12 µg) or anti-IgG Ab (12 ug, sc-2025, Santa Cruz Biotechonogy) for 18 hours. After washing with tris-ethylene diamine tetra acetic acid including of 1% of sulfuric acid dodecyl buffer, to reverse the cross-link, the immunoprecipitated DNA and control DNA samples were prepared using previously published methods [18]. Adaptor-ligand DNA fragments were size-fractionated in 2% agarose gel (E-Gel®, Invitrogen), and the 150–350 bp fraction was recovered. Each DNA fragment obtained was amplified by 15 cycles of PCR (PCR primer 1.1 and 2.1, Illumina®). Each ChIP DNA library (3.5 pM) was denatured and loaded onto the Illumina Flow Cell using the single-read cluster plate kit (v2, Illumina®) on the cBot (Illumina®) for cluster generation and sequenced using the Illumina Genome Analyzer (GAIIx, Illumina®) over 36 cycles with related SBS sequencing Reagents (v5, Illumina®). FASTQ files were generated from readings passing quality filters for further ChIP-data analysis.

### Data analysis for ChIP-seq, motif analysis, and the detection of candidate genes

Short-read sequences were aligned to human genome sequences (hg19 from UCSC Genome Browser; http://genome.uscs.edu/) using the DNAnexus program (https://dnanexus.com/). Peak-calling was based on the QuEST algorithm. We set a strict threshold for the analysis to improve data quality. Peaks with more than a 3-fold increase in signal intensity versus input DNA and with more than 30-fold increase in read count in sample compared to a uniform distribution of the same sample reads across the genome were detected. Within these, peaks with a q-value<10^−10^ (i.e. the q-score in −log_10_(q-score) is greater than 10) were accepted as statistically significant.

For motif analysis, binding site sequences were confirmed against consensus motif sequences using TRANSFAC® (BIOBASE, Germany).

Genes were identified as candidate genes for regulation by STAT5A and/or STAT5B if the gene existed within 10,000 bp from each detected binding site. Candidate genes were defined as “specifically detected genes” if they were detected by only anti-STAT5A Ab ChIP-seq (STAT5A ChIP-seq) or only anti-STAT5B Ab ChIP-seq (STAT5B ChIP-seq). Genes were defined as “dominantly detected genes” if there was more than a 2-fold difference in q-score between STAT5A ChIP-seq results and STAT5B ChIP-seq results. Genes were regarded as “equally detected genes” if q-scores differed by less than 2-fold between STAT5A ChIP-seq results and STAT5B ChIP-seq results, or were equivalent.

### Quantitative PCR

RNA was isolated from 2.0×10^5^ CD4^+^ T cells transfected with STAT5A, STAT5B, or control siRNA via RNeasy kits (Qiagen, Germany) according to the manufacturer's protocol. KD cells were prepared using previously published methods [16]. For cDNA synthesis, 500 ng of total RNA were transcribed with cDNA transcription reagents (Applied Biosystems, USA) using random hexamers, according to the manufacturer's protocol. Gene expression was measured in real time using primers (Invitrogen, Applied Biosystems). The expression levels of each gene were adjusted with β-glucuronidase as an internal control, and were compared with expression levels detected in the control siRNA samples. In addition, ΔΔCq (Cq for quantification cycle) method was used as a normalized determination of genes that were knocked down in the siRNA experimental controls. The data are shown as a ratio relative to each control sample; the levels of each control sample are indicated as 1. All PCR assays were performed with Sybr-Green-based technology (Sigma-Aldrich). QT-PCR results were analyzed via paired t-test using GraphPad Prism 6 (GraphPad Software, Inc.). P<0.05 was set as the threshold for statistical significance. “Specifically regulated gene” is biologically defined in this article as a gene that is transcriptionally-controlled by STAT5A but not STAT5B, and by STAT5B but not STAT5A. “Specifically regulated gene” is statistically defined here as if P<0.01 in only *STAT5A* KD or *STAT5B* KD CD4^+^ T cells compared to the control CD4^+^ T cells.

## Results

### Localization of STAT5A and STAT5B, and their dimerization

**Figure 1:**
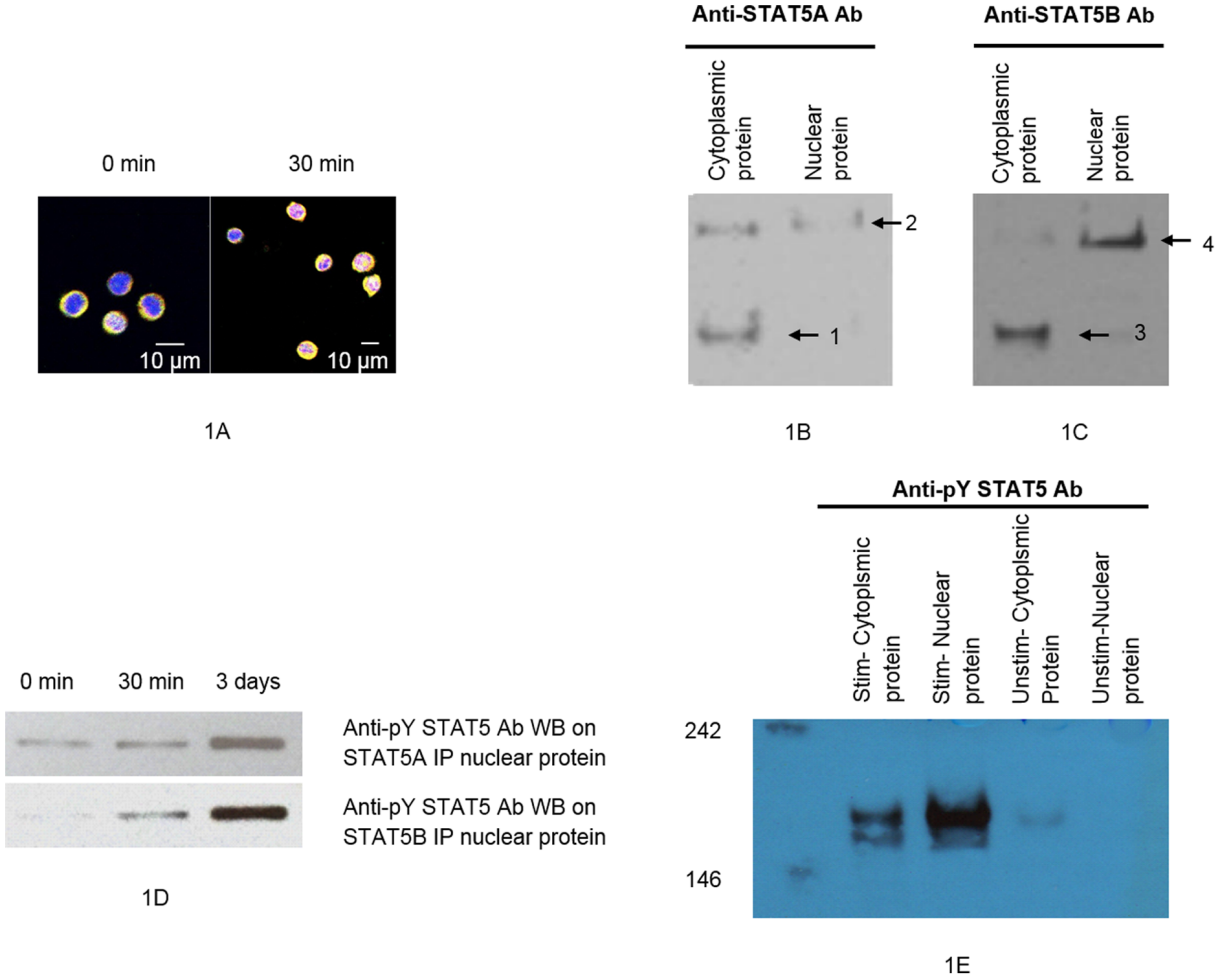
Localization of STAT5A and STAT5B, and monomers and dimers of STAT5A and STAT5B. **A** demonstrates translocation of STAT5A and STAT5B into cell nuclei after 30-2 (40× confocal). Yellow, STAT5A; purple, STAT5B; blue, nucleus. **B** and **C**. Detection of STAT5A and STAT5B proteins in cytoplasmic or nuclear proteins fractionated from CD4^+^ T cells after PHA-P stimulation for 3 days followed by incubation with rhIL-2 for 30 min. **B**. STAT5A monomer (91 kDa, arrow 1) and STAT5A dimer (arrow 2) in native cytoplasmic or nuclear proteins, detected with anti-STAT5A Ab. **C**. STAT5B monomer (90 kDa, arrow 3) and STAT5B dimer (arrow 4) in native cytoplasmic or nuclear proteins, detected using anti-STAT5B Ab. **D**. Detection of phosphorylated STAT5 proteins in STAT5A- or STAT5B- immunoprecipitated nuclear proteins fractionated from CD4^+^ T cells after PHA-P stimulation for 3 days followed by incubation with rh-IL-2 for 0 min, 30 min or 3 days. **E**. Detection of phosphorylated STAT5 proteins in cytoplasmic or nuclear proteins fractionated from CD4^+^ T cells after PHA-P stimulation for 3 days followed by incubation with rhIL-2 for 3 days, and control (unstimulated condition).

We first determined the kinetics of intracellular localization of STAT5A and STAT5B in CD4^+^ T cells at different time points after addition of rhIL-2. We found that nuclear translocation of both STAT5A and STAT5B was detectable as early as 30 min after addition of rhIL-2 (**Fig. 1A**). STAT5A and STAT5B monomers or dimers were detected in native cytoplasmic or nuclear proteins extracted from CD4^+^ T cells after 30 minutes in the presence of rhIL-2 (**Fig. 1B, 1C** **)**. This time point was therefore chosen for basic condition of ChIP-seq experiment. Additionally, the phosphorylated STAT5 proteins were detected in nuclear or cytoplasmic proteins extracted from CD4^+^ T cells after 30 min or 3 days in the presence of rhIL-2 (**Fig. 1D, 1E**).

### ChIP-seq analysis of human CD4^+^ T cells detected candidate genes potentially regulated by STAT5A and/or STAT5B

To detect candidate genes potentially regulated by STAT5A and/or STAT5B, we assessed DNA binding patterns via ChIP-seq analysis in human CD4^+^ T cells.

**Figure 2:**
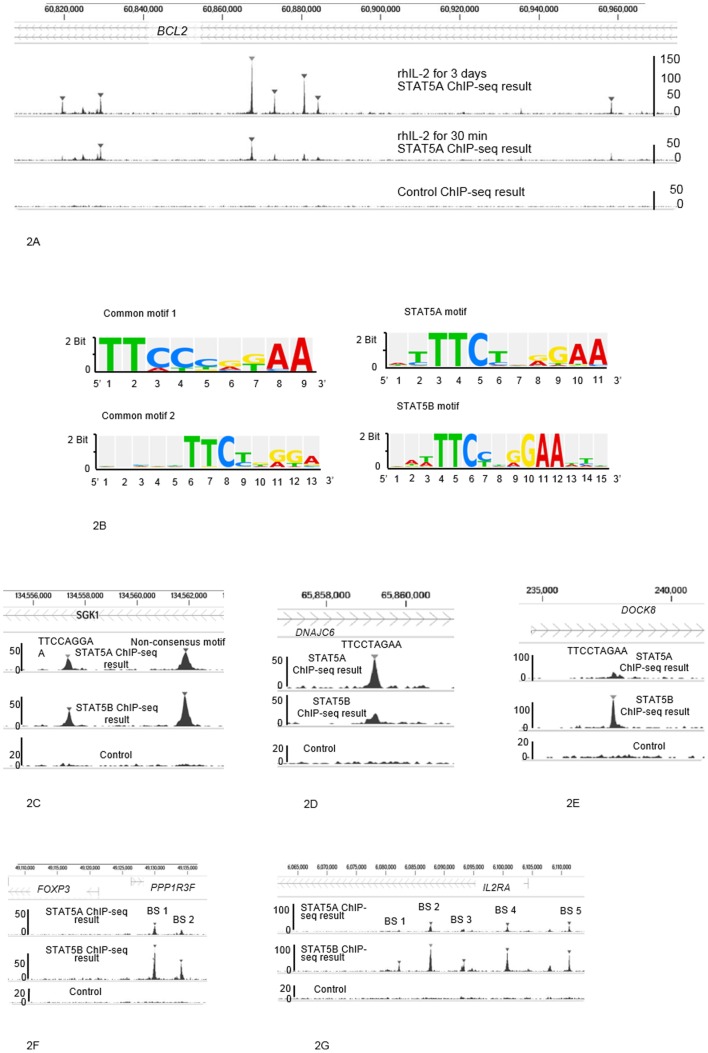
Binding ability, motif sequences and binding sites. **A** shows binding ability of STAT5A after 3 days of exposure to rhIL-2 versus binding ability of STAT5A after 30 min of exposure to rhIL-2. It shows results of STAT5A ChIP-seq on chromosome 18 performed in CD4^+^ T cells incubated with rhIL-2 for 3 days (top); in CD4^+^ cells incubated with rhIL-2 for 30 min (middle); compared with control ChIP-seq in CD4^+^ T cells (bottom). **B** shows consensus motif sequences for STAT5A and/or STAT5B. **C** shows binding sites for *SGK1*, detected by both STAT5A and STAT5B ChIP-seq. **D** and **E** show the detection of the sequence “TTCCTAGAA” by STAT5A ChIP-seq in *DNAJC6*, and by STAT5B ChIP-seq in *DOCK8*. **F** shows that *FOXP3* and *PPP1R3F* are located within 10,000 bp of the two binding sites. **G** shows the five binding sites for *IL2RA*.

Following Liao *et al.*'s report of different binding abilities for *Il4ra* by Stat5a and Stat5b in mouse CD4^+^ T cells following different lengths of exposure to rhIL-2 [12], we tested whether different binding abilities of STAT5A and STAT5B in human CD4^+^ T cells were dependent on duration of rhIL-2 exposure. We found that when PHA-P-activated CD4^+^ T cells were cultured in the presence of rhIL-2 for 30 min, STAT5A ChIP-seq detected 245 binding sites, and STAT5B ChIP-seq detected 248 binding sites. When PHA-P activated CD4^+^ T cells were exposed to rhIL-2 for 3 days, however, STAT5A ChIP-seq detected 908 binding sites, and STAT5B ChIP-seq detected 1286 binding sites (**Fig. 2A**). This indicates that longer exposure to rhIL-2 leads to increased changes in gene regulation. We therefore decided to focus our studies on results obtained after 3 days of rhIL-2 exposure.

Candidate genes were divided into 5 groups: genes detected by both STAT5A and STAT5B ChIP-seq (common), 273; genes detected only by STAT5A ChIP-seq (STAT5A-specific), 33; genes detected by STAT5A ChIP-seq, with a greater than 2-fold difference in q-score (STAT5A-dominant), 41; genes detected only by STAT5B ChIP-seq (STAT5B-specific), 187; genes detected by STAT5B ChIP-seq, with a greater than 2-fold difference in q-score (STAT5B-dominant), 146. The genes in each group are listed in **Table S1**.

**Table 1**, **2** and **3** list the genes and their binding sequences identified as being of greatest interest by ChIP-seq. 9, 10, and 8 genes were chosen for the common, STAT5A- or STAT5B-specific, and STAT5A- or STAT5B-dominant groups, respectively. The 9 genes chosen for the common group had the smallest differences in q-score between STAT5A and STAT5B ChIP-seq. The 10 genes in the specific groups had the largest absolute q-scores in either the STAT5A (4) or the STAT5B (6) ChIP-seq results. The dominant group comprised 8 genes (4 STAT5A-dominant, 4 STAT5B-dominant) with the largest differences in q-score between STAT5A and STAT5B ChIP-seq.

**Table 1:** Detected binding site sequences on candidate genes detected equally by both STAT5A and STAT5B ChIP-seq.

Gene	Sequence of Binding Site 1	Sequence of Binding Site 2	Sequence of Binding Site 3
	TTCCAGGAA	TTCCCCAGAA	
*SGK1*	Chr 6: 134,557,320–134,557,328	Chr 6 : 13,561,874–13,561,883	
	TTCCAAGAA		
*GTF2H5*	Chr 6: 158,628,727–158,628,732		
	TTCTAAGAA		
*BCL2L1*	Chr 20: 30,263,758–30,263,766		
	TTCTTAAA		
*SLC22A5*	Chr 5: 131,732,692–131,732,700		
	TTCTTGGAA		
*CDKAL1*	Chr 6: 20,534,571–20,534,579		
	TTCCTGGAA		
*DNM2*	Chr 19: 10,908,448–10,908,456		
	TTCTCAGAA	TTCTTGAAA	
*MBP*	Chr 18: 74,779,503–74,779,511	Chr 18: 74,814,691–74,814,699	
	TTCTAGGAA	TTCCCTGAA	TTCTTAGAA
*DUSP5*	Chr 10: 112,261,216–112,261,224	Chr 10: 112,262,968–112,262,976	Chr 10: 112,264,047–112,264,055
	TTCTGAAAA		
*ARL4C*	Chr 2: 235,399,028–235,399,036		

**Table 2:** Detected binding site sequences on candidate genes detected specifically or dominantly by STAT5A ChIP-seq.

Gene	Sequence of Binding Site 1
	TTCCTGGAA
*NDRG1* (sp)	Chr 8: 134,315,482–134,315,490
	TTCCTAGAA
*DNAJC6* (sp)	Chr 1: 65,859,207–65,859,215
	TTCCTGGAA
*CBS* (sp)	Chr 21: 44,465,709–44,465,717
	TTGCTATGAA
*PPP2R2B* (sp)	Chr 5: 146,174,088–146,174,097
	TTCCAGGAA
*ST3GAL1* (dom)	Chr 8: 134,532,952–134,532,960
	TTCTTGGAA
*SAMD4A* (dom)	Chr 14: 55,240,186–55,240,194
	TTCTCAGAA
*SSH2* (dom)	Chr 17: 28,087,490–28,087,498
	TTCTTGTAA
*MAP3K5* (dom)	Chr 6: 137,072,913–137,072,921

**Abbreviation**: sp, specifically detected; dom, dominantly detected.

**Table 3:** Detected binding site sequences on candidate genes detected specifically or dominantly with STAT5B ChIP-seq.

Gene	Sequence of Binding Site 1 (or 4)	Sequence of Binding Site 2 (or 5)	Sequence of Binding Site 3
	TTCCTAGAA		
*DOCK8* (sp)	Chr 9: 237,726–237,734		
	AAGCTT		
*SNX9* (sp)	Ch 6: 158,281,655–158,281,660		
	TTCTCAGAA		
*LNPEP* (sp)	Chr 5: 96,294,057–96,294,065		
	TTCATGGCAGATGAA		
*SKAP1* (sp)	Ch 17: 46,271,592–46,271,606		
	TTAGTGGAA		
*PTGER1* (sp)	Chr 14: 52,786,605–52,786,613		
	TCCAGGAA		
*DIDO1* (sp)	Chr 20: 61,549,907–61,549,914		
	TTCCAAGAA		
*TNFSF10* (dom)	Chr 3: 172,235,834–172,235,842		
	CAGCTCTT	TTCTAAGAA	
*FOXP3* (dom)	Chr X: 49,129,970–49,129,981	Chr X: 49,134,028–49,134,036	
	TTCTAAGAA	ACAGTCTT	TTCAAACGAA
*IL2RA* (dom)	Chr 10: 6,082,278–6,082,286	Chr X: 6,087,689–6,087,697	Chr X: 6,093,331–6,093,340
	TTCTGAGAA	TTCTACGAA	
	Chr X: 6,100,740–6,100,752	Chr X: 6,111,306–6,111,314	
	TTCTTTGAA		
*UGCG* (dom)	Chr 9: 114,660,802–114,660,810		

**Abbreviation**: sp, specifically detected; dom, dominantly detected.

### DNA binding sequences within candidate genes detected via ChIP-seq

To verify the binding sequences detected with ChIP-seq, we compared them to previously established consensus motif sequences for STAT5A and/or STAT5B. 30 of the 36 binding sequences detected by STAT5A and/or STAT5B ChIP-seq were in accordance with consensus motif sequences (**Table 1**, **2** and **3**; **Fig. 2B**). The remaining 6 binding sequences, despite not aligning with consensus motif sequences, contained the core STAT5 consensus motif sequence “TTC-GAA” [19], TTC- or -GAA.

Several DNA binding sequences were shared between candidate genes (**Tables 1**, **2** and **3**). The sequence “TTCCAGGAA” was detected equally by both STAT5A and STAT5B ChIP-seq in *SGK1*, as well as dominantly by STAT5A ChIP-seq in *ST3GAL1* (**Table 1**, **2** and **4**; **Fig. 2C**). The sequence “TTCCAAGAA” was detected equally by both STAT5A and STAT5B ChIP-seq in *GTF2H5*, but dominantly by STAT5B ChIP-seq in *TNFSF10* (**Table 1**, **3** and **4**). The sequence “TTCTAAGAA” was detected equally by both STAT5A and STAT5B ChIP-seq in *BCL2L1*, and detected dominantly by STAT5B ChIP-seq in *FOXP3* and *IL2RA* (**Table 1**, **3** and **4**). The sequence “TTCTTGGAA” was detected equally by both STAT5A and STAT5B ChIP-seq in *CDKAL1* and detected dominantly by STAT5A ChIP-seq in *SAMD4A*. The sequence “TTCCTGGAA” was detected equally by both STAT5A and STAT5B ChIP-seq in *DNM2* and detected specifically by STAT5A ChIP-seq in *NDRG1* (**Table 1**, **2** and **4**). The sequence “TTCTCAGAA” was detected equally by both STAT5A and STAT5B ChIP-seq in *MBP*, but detected dominantly by STAT5A ChIP-seq in *SSH2* and specifically by STAT5B ChIP-seq in *LNPEP*. The sequence “TTCCTAGAA” was detected specifically by STAT5A ChIP-seq on *DNAJC6*, and was also detected specifically by STAT5B ChIP-seq on *DOCK8* (**Table 2** and **3**; **Fig. 2D** and **2E**).

**Table 4:** Binding site q-scores for STAT5A and STAT5B ChIP-seq.

	Gene name	q-score of Binding Site 1 (or 4)		q-score of Binding Site 2 (or 5)		q-score of Binding Site 3	
		STAT5A ChIP-seq	STAT5B ChIP-seq	STAT5A ChIP-seq	STAT5B ChIP-seq	STAT5A ChIP-seq	STAT5B ChIP-seq
E	*SGK1*	339	328	79	75		
E	*GTF2H5*	304	288				
E	*BCL2L1*	279	405				
E	*SLC22A5*	403	394				
E	*CDKAL1*	695	603				
E	*DNM2*	463	475				
E	*MBP*	303	389	13	20		
E	*DUSP5*	953	1040	95	142	146	146
E	*ARL4C*	516	583				
5A	*NDRG1* (sp)	285	–				
5A	*DNAJC6* (sp)	283	–				
5A	*CBS* (sp)	236	–				
5A	*PPP2R2B* (sp)	223	–				
5A	*ST3GAL1* (dom)	339	23				
5A	*SAMD4A* (dom)	428	18				
5A	*SSH2* (dom)	618	82				
5A	*MAP3K5* (dom)	237	26				
5B	*DOCK8* (sp)	–	621				
5B	*SNX9* (sp)	–	535				
5B	*LNPEP* (sp)	–	448				
5B	*SKAP1* (sp)	–	499				
5B	*PTGER1* (sp)	–	446				
5B	*DIDO1* (sp)	–	416				
5B	*TNFSF10* (dom)	58	633				
5B	*FOXP3* (dom)	456	1006	–	42		
5B	*IL2RA* (dom)	–	24	120	301	–	22
		120	245	74	154		
5B	*UGCG* (dom)	73	949				

E indicates a group of the candidate genes detected equally by STAT5A and STAT5B ChIP-seq. 5A indicates a group of the candidate genes detected specifically or dominantly by STAT5A ChIP-seq. 5B indicates a group of the candidate genes detected specifically or dominantly by STAT5B ChIP-seq. (-) indicates no significant detection.

Because ChIP-seq detected multiple binding sites for *SGK1*, *MBP*, and *DUSP5*, we evaluated whether each binding site was equally detected by STAT5A and STAT5B ChIP-seq, and found this to be the case (**Table 4**).

**Figure 3:**
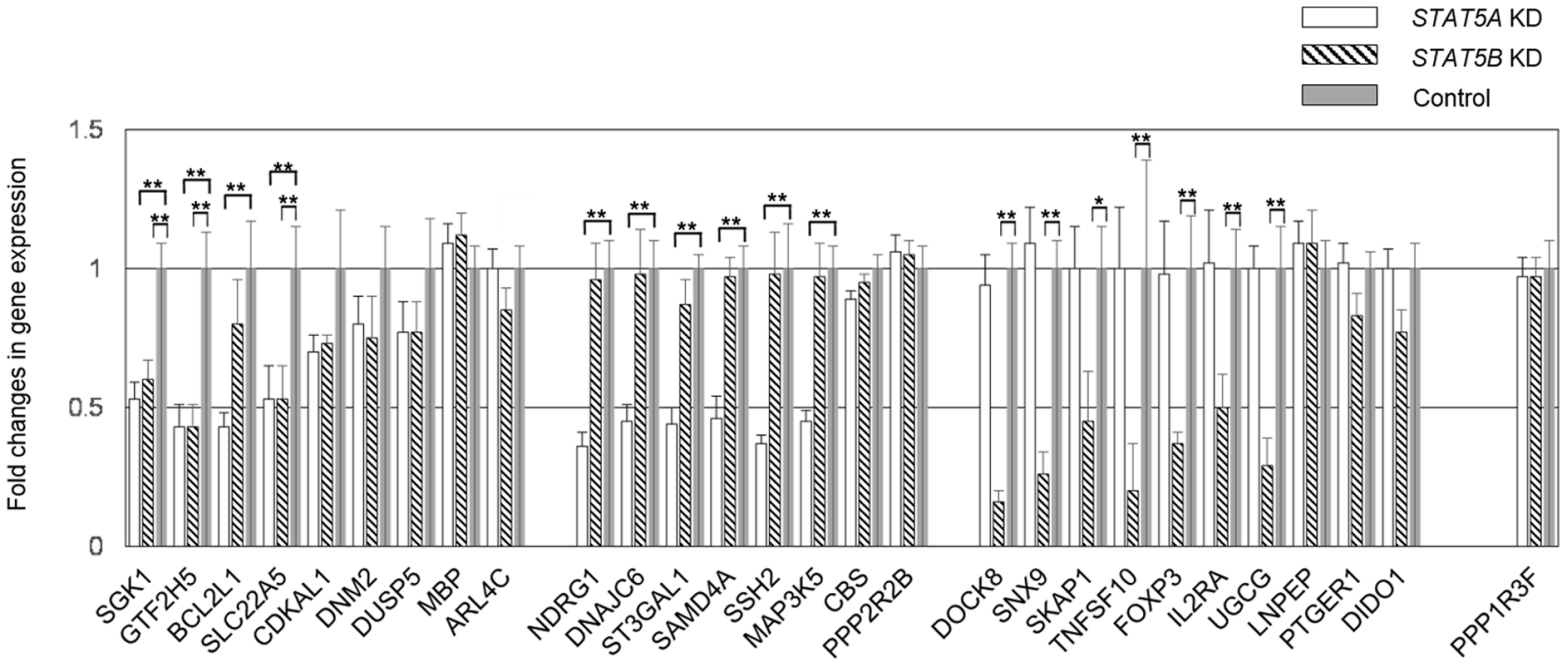
Validation of candidate genes from ChIP-seq via QT-PCR in *STAT5A* and *STAT5B* knock-down human CD4^+^ T cells. The expression levels were compared *STAT5A* KD or *STAT5B* KD CD4^+^ T cells with control CD4^+^ T cells. Genes regulated similarly by both STAT5A and STAT5B are *SGK1*, *GTF2H5* and *SLC22A5*. Genes regulated specifically by STAT5A are *NDRG1*, *DNAJC6*, *ST3GAL1*, *SAMD4A*, *SSH2*, *MAP3K5* and *BCL2L1*. Genes regulated specifically by STAT5B are *DOCK8*, *SNX9*, *SKAP1*, *TNFSF10*, *FOXP3*, *IL2RA* and *UGCG*. Data is presented as mean ± SEM. *, P<0.01; **, P<0.001. There were no statistical differences if not annotated. **Abbreviations**: *SGK1*, serum/glucocorticoid regulated kinase 1; *GTF2H5*, general transcription factor IIH, polypeptide 5; *BCL2L1*, BCL2-like 1; *SLC22A5*, solute carrier family 22 (organic cation/carnitine transporter), member 5; *CDKAL1*, CDK5 regulatory subunit associated protein 1-like 1; *DNM2*, dynamin 2; *DUSP5*, dual specificity phosphatase 5; *MBP*, myelin basic protein; *ARL4C*, ADP-ribosylation factor-like 4C; *NDRG1*, N-myc downstream regulated 1; *DNAJC6*, DnaJ (Hsp40) homolog, subfamily C, member 6; *ST3GAL1*, ST3 beta-galactoside alpha-2,3-sialyltransferase 1; *SAMD4A*, sterile alpha motif domain containing 4A; *SSH2*, slingshot protein phosphatase 2; *MAP3K5*, mitogen-activated protein kinase kinase kinase 5; *CBS*, cystathionine-beta-synthase; *PPP2R2B*, protein phosphatase 2, regulatory subunit B, beta; *DOCK8*, dedicator of cytokinesis 8; *SNX9*, sorting nexin 9; *SKAP1*, src kinase associated phosphoprotein 1; *PTGER1*, prostaglandin E receptor 1 (subtype EP1), 42 kDa; *DIDO1*, death inducer-obliterator 1; *TNFSF10*, tumor necrosis factor (ligand) superfamily, member 10; *FOXP3*, forkhead box P3; *IL2RA*, interleukin 2 receptor, alpha; *UGCG*, UDP-glucose ceramide glucosyltransferase; *LNPEP*, leucyl/cystinyl aminopeptidase; *PPP1R3F*, protein phosphatase 1, regulatory subunit 3F.

Similarly, because there were multiple binding sites for both *FOXP3* and *IL2RA*, we evaluated whether each binding site was detected specifically or dominantly by STAT5B ChIP-seq. First, we identified the gene regulated by the binding site of chr X 49,129,970–49,129,981 and 49,134,028–49,134,036 (**Table 3**, **Fig. 2F**) because two genes, *FOXP3* and *PPP1R3F*, are located within 10,000 bp of the binding sites. QT-PCR showed these binding sites corresponded to *FOXP3*, as expression levels of *PPP1R3F* remained unaffected in both *STAT5A* and *STAT5B* KD human CD4^+^ T cells (**Fig. 3**; P = 0.50 respectively), while *FOXP3* expression levels were relatively decreased as compared to controls in *STAT5B* KD human CD4^+^ T cells (**Fig. 3**; P<0.001). Both binding sites for *FOXP3* were detected (**Table 3**, **Fig. 2F**) by STAT5B ChIP-seq (**Table 4**). Five binding sites for *IL2RA* were detected (**Table 3**, **Fig. 2G**) either dominantly or specifically by STAT5B ChIP-seq (**Table 4**).

### Validation of candidate genes by QT-PCR in *STAT5A* or *STAT5B* KD human CD4^+^ T cells

To confirm whether the candidate genes were regulated by STAT5A and/or STAT5B, we measured expression levels of each gene (listed in **Table 1**, **2** and **3**) via QT-PCR in *STAT5A* or *STAT5B* KD human CD4^+^ T cells. When compared with control siRNA-transfected CD4^+^ T cells, STAT5A siRNA-transfected human CD4^+^ T cells showed a 45% reduction in *STAT5A* gene expression as compared to control after 3 days (P<0.001); STAT5B siRNA-transfected human CD4^+^ T cells showed a 58% reduction in *STAT5B* gene expression as compared to control after 3 days (P<0.001) [16]. There was no significant difference in KD ratio between *STAT5A* and *STAT5B* (P = 0.20).

Expression levels of *SGK1*, *GTF2H5* and *SLC22A5* decreased similarly in *STAT5A* and *STAT5B* KD CD4^+^ T cells versus control CD4^+^ T cells, whereas no significant changes in expression levels of *CDKAL1*, *DNM2*, *DUSP5*, *MBP* and *ARL4C* were seen between KDs and control CD4^+^ T cells (**Fig. 3**). Expression levels of *NDRG1*, *DNAJC6*, *ST3GAL1*, *SAMD4A*, *SSH2*, *MAP3K5* and *BCL2L1* were specifically decreased in *STAT5A* KD CD4^+^ T cells versus control CD4^+^ T cells (i.e., P<0.01 in only *STAT5A* KD CD4^+^ T cells, **Fig. 3**). There were no significant differences in the expression levels of these genes between *STAT5B* KD CD4^+^ T cells and control CD4^+^ T cells. Expression levels of *DOCK8*, *SNX9*, *SKAP1*, *TNFSF10*, *FOXP3*, *IL2RA*, and *UGCG* were specifically decreased in *STAT5B* KD CD4^+^ T cells versus control CD4^+^ T cells (i.e., P<0.01 in only *STAT5B* KD CD4^+^ T cells, **Fig. 3**). There were no significant differences in the expression levels of these genes between *STAT5A* KD CD4^+^ T cells and control CD4^+^ T cells. Finally, no significant changes were seen in the expression levels of *CBS*, *PPP2R2B*, *LNPEP PTGER1* and *DIDO1* in either *STAT5A* or *STAT5B* KD CD4^+^ T cells versus control CD4^+^ T cells.

As was seen with ChIP-seq, genes sharing binding sequences did not always display identical patterns of expression in *STAT5A* and *STAT5B* KD CD4^+^ T cells. Genes sharing binding sequences but with differing behaviors were: *SGK1* (decreased similarly in both KDs) and *ST3GAL1* (decreased specifically in *STAT5A* KD, no change in *STAT5B* KD); *GTF2H5* (decreased similarly in both KDs) and *TNFSF10* (deceased specifically in *STAT5B* KD, no change in *STAT5A* KD); *BCL2L1* (decreased specifically in *STAT5A* KD, no change in *STAT5B* KD), *FOXP3* and *IL2RA* (decreased specifically in *STAT5B* KD, no change in *STAT5A* KD); *CDKAL1* (no change in both KDs) and *SAMD4A* (decreased specifically in *STAT5A* KD, no change in *STAT5B* KD); *DNM2* (no change in both KDs) and *NDRG1* (decreased specifically in *STAT5A* KD, no change in *STAT5B* KD); *MBP* and *LNPEP* (no change in both KDs) and *SSH2* (decreased specifically in *STAT5A* KD, no change in *STAT5B* KD); and *DNAJC6* (decreased specifically in *STAT5A* KD, no change in *STAT5B* KD) and *DOCK8* (decreased specifically in *STAT5B* KD, no change in *STAT5A* KD).

Known roles of the genes are summarized in **Table 5**, **6** and **7**.

**Table 5:** Genes associated with regulation by STAT5A and STAT5B by QT-PCR.

Gene	Full name	Location	Roles
*SGK1*	serum/glucocorticoid regulated kinase 1	6q23	Activation of certain potassium, sodium and chloride channels and regulation of inflammatory cell proliferation and apoptosis [39].
*GTF2H5*	general transcription factor IIH, polypeptide 5	6q25.3	Encodes a subunit of transcription/repair factor TFIIH, which functions in gene transcription and DNA repair. Mutations in this gene cause DNA repair-deficient trichothiodystrophy [40].
*SLC22A5*	solute carrier family 22 (organic cation/carnitine transporter), member 5	5q23.3	Involved in the active cellular uptake of carnitine. Mutations of this gene cause systemic primary carnitine deficiency [41].

**Table 6:** Genes associated with regulation by STAT5A by QT-PCR.

Gene	Full name	Location	Roles
*NDRG1*	N-myc downstream regulated 1	8q24.3	Involved in hypoxic stress and androgen hormone response, cell growth and differentiation, and apoptosis [42]. NDRG1 deficiency causes Schwann cell dysfunction and is a cause of Charcot-Marie-Tooth disease type 4D, which is characterized by motor and sensory nerve dysfunction [43].
*DNAJC6*	DNAJ (Hsp40) homolog, subfamily C, member 6	1p31.3	Involved in recycling of synaptic vesicles in neurons [44].
*ST3GAL1*	ST3 beta-galactoside alpha-2,3-sialyltransferase 1	8q24.22	When inactivated, renders CD8^+^, but not CD4^+^, T cells susceptible to apoptosis [45].
*SAMD4A*	sterile alpha motif domain containing 4A	14q22.2	Expressed during synaptogenesis; modulates synapse formation [46].
*SSH2*	slingshot protein phosphatase 2	17q11.2	Critical for neurite extension through actin dynamics [47].
*MAP3K5*	mitogen-activated protein kinase kinase kinase 5	6q22.33	Contributes to apoptosis of plasma cells [48].
*BCL2L1*	BCL2-like 1	20q11.21	Prevents apoptosis [49].

**Table 7:** Genes associated with regulation by STAT5B by QT-PCR.

Gene	Full name	Location	Roles
*DOCK8*	dedicator of cytokinesis 8	9p24.3	Critical and intrinsic to peripheral CD8^+^ T cell survival and function [32]. Mutations in this gene result in the autosomal recessive form of hyper-IgE syndrome [33].
*SNX9*	sorting nexin 9	6q25.1-q26	Subunit of WASPs (Wiskott-Aldrich syndrome protein)/SNX9/p85/CD28, which enables signal transduction pathway required for CD28-mediated T cell costimulation [35].
*SKAP1*	src kinase associated phosphoprotein 1	17q21.32	SKAP-55 regulates integrin-mediated adhesion and conjugate formation between T cells and antigen-presenting cells [50].
*TNFSF10*	tumor necrosis factor (ligand) superfamily, member 10	3q26	This protein is a member of TNF family of cytokines, which are structurally related proteins playing important roles in regulating cell death, immune response, and inflammation [51].
*FOXP3*	forkhead box P3	Xp11.23	Crucial for Treg development and function. Defects in this gene cause immunodeficiency polyendocrinopathy, enteropathy, X-linked syndrome (IPEX) [52].
*IL2RA*	interleukin 2 receptor, alpha	10p15-p14	Constitutes the alpha chain of the high-affinity IL2 receptor [53].
*UGCG*	UDP-glucose ceramide glucosyltransferase	9q31	When silenced, leads to p53-dependent apoptosis [54]. Prolonged overabundance of glucosylceramide is detrimental, as is seen in Gaucher disease [55].

## Discussion

This study is the first to demonstrate, to our knowledge, redundant and non-redundant roles for STAT5A and STAT5B in human gene regulation. Genome-wide identification of STAT5A and STAT5B target genes in human CD4^+^ T cells yielded a number of candidate genes, some of which appeared to be regulated by both STAT5A and STAT5B, while others were associated with regulation by either STAT5A or STAT5B. ChIP-seq results were further tested for validity by QT-PCR in *STAT5A* or *STAT5B* KD CD4^+^ T cells, which identified several genes whose expression was regulated by STAT5A and/or STAT5B. Both STAT5A and STAT5B regulated genes implicated in cell proliferation and apoptosis, gene transcription and DNA repair, and the active cellular uptake of carnitine. STAT5A was associated with non-redundant (i.e., not shared with STAT5B) roles in regulating genes relevant to neurite extension, synaptic vesicle recycling, anti-apoptosis, hypoxic stress, and androgen response; STAT5B was linked to non-redundant (i.e., not shared with STAT5A) roles in regulating genes important to IgE production, peripheral CD8^+^ T cell survival and function, Treg development and function, and T cell activation and proliferation. Overall, STAT5B was therefore highly associated with regulation of genes associated with T cell-specific functions, while STAT5A appeared to interact with genes known to play major roles in neural development and function.

It has been suggested that structural differences between STAT5A and STAT5B DNA-binding domains, C-termini (which contain transactivation domains), and N-termini (which mediate oligomerization) may result in non-redundant roles in each gene's regulation. Our results supported this hypothesis, showing different binding behaviors between STAT5A and STAT5B to genes with differing binding sites: for example, *MAP3K5* (STAT5A ChIP-seq, STAT5A-dominant; QT-PCR, regulated specifically by STAT5A), *SNX9* (STAT5B ChIP-seq, STAT5B-specific; QT-PCR, regulated specifically by STAT5B), and *UGCG* (STAT5B ChIP-seq, STAT5B-dominant; QT-PCR, regulated specifically by STAT5B).

At the same time, we found that binding site sequences alone could not predict STAT5A versus STAT5B binding behavior, observing several gene-specific, but not sequence-specific, differences in STAT5A and STAT5B binding. For example, the same binding site sequence was detected within the gene *SGK1* by both STAT5A and STAT5B ChIP-seq, but was dominantly detected by STAT5A ChIP-seq in *ST3GAL1*. After eliminating experiment-specific limitations such as differential affinity between anti-STAT5A and anti-STAT5B Abs, this discrepancy suggests that co-activators may also be important in establishing differential binding and transcriptional activities between STAT5A and STAT5B. This is supported by previous findings that Crkl, a STAT5B- and Stat5b-binding co-activator, significantly increases STAT5B and Stat5b binding to their target genes in humans and mice, respectively [20], [21], and that centrosomal P4.1-associated protein augments STAT5 mediated transcriptional activity in humans [22], though this has not been reported in rodents.

Alternatively, the following mechanisms have been proposed for STAT protein regulation of target genes: (1) binding to DNA binding sites to directly drive transcription; (2) forming transcriptional complexes with non-STAT transcription factors to trigger transcription through a STAT; (3) interaction with non-STAT DNA binding sites; and (4) cooperation between STATs and non-STAT transcription factors to activate transcription via binding to clustered independent DNA binding sites [23].

It is also possible that the identified binding sites are not linked to the assigned candidate genes but are regulating further distant genes, as a binding site for STAT5 was found over 10,000 bp away from the target gene [6]. Additionally, the magnitude of opportunistic genomic STAT5 binding does not necessarily translate into transcriptional activation of neighboring genes. For example, Zhu et al. demonstrated that STAT5 binding to promoter upstream sequences does not automatically convey STAT5 control over those genes [24]. Yamaji et al. also reported that STAT5 binding to promoter sequences was not necessarily an indicator for the overall expression of the respective genes [25].

These mechanisms may help explain the discrepancies seen between our ChIP-seq and QT-PCR results, in which several genes associated with STAT5A and/or STAT5B by ChIP-seq (*CDKAL1*, *DNM2*, *DUSP5*, *MBP*, *ARL4C*, *CBS*, *PPP2R2B*, *LNPEP*, *PTGER1* and *DIDO1*) showed no change in expression levels in QT-PCR performed in *STAT5A* or *STAT5B* KD CD4^+^ T cells when compared to control CD4^+^ T cells.

Our finding that STAT5A and STAT5B are associated with redundant roles in cell proliferation and apoptosis is in keeping with several reports showing the potential of STAT5 to function as an oncogene or a tumor suppressor in humans [26]. *SGK1*, identified in our study as a target gene of both STAT5A and STAT5B, has also been implicated in the pathophysiology of non-small-cell lung cancer and adrenocortical tumors in humans [27], [28], although this has not been reported in rodents.

Our study demonstrates that the majority of the genes associated with STAT5A-specific regulation are related to neural development and function. However, STAT5A-deficient patients have yet to be reported. One might therefore speculate that STAT5A mutations may be lethal in humans, or that they may be involved in neurodevelopmental disorders that have yet to be associated with STAT5A dysfunction. Indeed, it has been reported that STAT5 is required for *in vitro* human neural development and function [29]. Additionally, prolactin, which stimulates both STAT5A and STAT5B with differing effects in humans [30], has been found to promote human neural stem cell proliferation through the JAK2-STAT5 pathway [31].

We found some genes to be specifically associated with regulation by STAT5B, and not STAT5A (i.e. *DOCK8*, *SNX9*, *FOXP3* and *IL2RA*). These genes may provide explanations for the phenotype of STAT5B-deficient patients (**Table 8**). For example, *DOCK8* is critical to IgE production and to the survival and function of peripheral CD8^+^ T cells in humans and mice [32]. DOCK8 deficiency results in autosomal recessive hyper-IgE syndrome with severe allergic manifestations, characterized by severe eczema, recurrent skin infection, mucocutaneous candidiasis, elevated serum IgE levels, and eosinophilia [33]. These characteristics align with previous reports from our group of increased serum IgE levels, decreased CD8^+^ T cell number, and severe eczema in STAT5B-deficient patients [11], [34], and suggest these characteristics of STAT5B deficiency may be related to downregulation of *DOCK8* in the absence of STAT5B. *SNX9* (sorting nexin 9) encodes subunits of WASp (Wiskott-Aldrich syndrome protein)/SNX9/p85/CD28, a complex involved in signal transduction of CD28-mediated T cell co-stimulation [35]. *SNX9* suppression may be a potential cause of impaired function and decreased number of T cells in STAT5B-deficient humans and mice.

**Table 8:** Phenotypic characteristics of STAT5B-deficient patients and potentially related genes, as detected via ChIP-seq and QT-PCR.

Phenotypic characteristic	Potentially related gene(s)
Serum IgE level elevation	*DOCK8*
Normal CD4^+^/CD8^+^ T cell ratio (Low CD4^+^ and CD8^+^ T cell number)	*IL2RA*, *DOCK8*, *SNX9*
Decreased Treg number	*FOXP3*, *IL2RA*
Low T cell number	*IL2RA*, *SNX9*

Our results also suggest differential gene expression of *STAT5A* and *STAT5B* occurs in human vs. mouse. For example, in humans, *IL2RA* is controlled only by STAT5B, not STAT5A; however, murine *IL-2α* is regulated by both Stat5a and Stat5b [36]. Furthermore, STAT5B-deficient patients show normal STAT5A expression and the phenotype similar to that of IL2RA-deficient patients [14], [15], [37], [38].

In conclusion, our data demonstrate potential redundant and non-redundant roles for STAT5A and STAT5B in human gene regulation, highlighting novel specific interactions that could not have been deduced merely from available data in the murine system. Differences between STAT5A and STAT5B DNA binding domains, C-termini, and N-termini were not always associated with differences in ability of STAT5A and STAT5B to bind their targets, suggesting one or more additional mechanisms may be important for establishing differential binding and transcription behaviors of STAT5A and STAT5B. We found redundant roles for STAT5A and STAT5B with genes associated with cell proliferation and apoptosis, and non-redundant roles with genes associated with neural development and function (STAT5A), and genes implicated in T cell development and function (STAT5B).

The elucidation of the roles of STAT5A and STAT5B furthers our understanding of potential mechanisms in cancer pathophysiology, neural disorders, and abnormal T cell immune function providing potential new targets to study in these diseases.

## Supporting Information

Figure S1 **Validation of anti-STAT5A Ab and anti-STAT5B Ab by Western blot.**
(TIF)

Table S1 **The list of candidate genes detected by STAT5A and/or STAT5B, equally, dominantly and specifically.**
(DOCX)
