# Supplementary figures and images for: Identification of STAT5A and STAT5B Target Genes in Human T Cells
Source: PLoS One. 2014 Jan 30;9(1):e86790. doi: 10.1371/journal.pone.0086790 (PMC3907443; doi:10.1371/journal.pone.0086790)

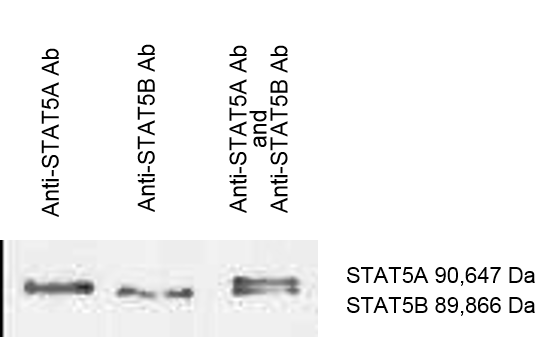

Supplement: Figure S1 — Validation of anti-STAT5A Ab and anti-STAT5B Ab by Western blot. (TIF) [file pone.0086790.s001.tif]
