# Supplementary material for: Identification of STAT5A and STAT5B Target Genes in Human T Cells
Source: PLoS One. 2014 Jan 30;9(1):e86790. doi: 10.1371/journal.pone.0086790 (PMC3907443; doi:10.1371/journal.pone.0086790)
Supplement: Table S1 — The list of candidate genes detected by STAT5A and/or STAT5B, equally, dominantly and specifically. (DOCX) [file pone.0086790.s002.docx]

**Supplementary information**

**Identification of STAT5A and STAT5B Target Genes in Human T cells**

Takahiro Kanai, M.D., Ph.D.^1*^, Scott Seki, BSc.^1^, Jennifer A Jenks, BSc.^1^, Arunima Kohli, BSc.^1^, Trupti Kawli, Ph. D^2^, Dorrelyn Patacsil Martin^2^, Michael Snyder, Ph. D^2^, Bacchetta Rosa, M.D^1,3^, Kari C Nadeau, M.D., Ph.D^1^.

^1^ Division of Immunology and Allergy, Department of Pediatrics, School of Medicine, Stanford University, Stanford, CA, USA.

^2^ Department of Genetics, School of Medicine, Stanford University, Stanford, USA.

^3^ San Raffaele Telethon Institute for Gene Therapy (HSR-TIGET), Milan, Italy

**# Correspondence to**: Takahiro Kanai, M.D., Ph.D.

**Address to**: Division of Immunology and Allergy, Department of Pediatrics, School of Medicine, Stanford University, Stanford, CA, 94305, USA.

**Phone**: +1-650-498-6865, **Fax**: +1-650-498-6865, **Email addresses**: tkanai@stanford.edu

**Supplemental Table 1.** The list of candidate genes detected by STAT5A and/or STAT5B, equally, dominantly and specifically

| **Equally detected genes** | | chr2 | *ARHGAP25* | **chr4** | *RBM47* | chr6 | *PNRC1* |
| --- | --- | --- | --- | --- | --- | --- | --- |
| **chr1** | *ATP6V1G3* |  | *KLF7* |  | *AFAP1* |  | *UTRN* |
|  | *CA6* |  | *DNAH6* |  | *LEF1* |  | *NHSL1* |
|  | *C1orf144* |  | *ICOS* |  | *TXK* |  | *UST* |
|  | *USP24* |  | *RNF144A* |  | *PPP3CA* |  | *C6orf204* |
|  | *PDE4B* |  | *KDM3A* |  | *UBE2K* |  | *TAGAP* |
|  | *YARS* |  | *ITGA6* |  | *ENPEP* |  | *ZNF390* |
|  | *PIK3CD* |  | *STAT4* |  | *RHOH* |  | *ZSCAN23* |
|  | *PGM1* |  | *RBMS1* |  | *ACOT7L* (or *N4BP2*) |  | *AIM1* |
|  | *CDC14A* |  | *YWHAQ* |  |  |  |  |
|  | *VAMP4* |  | *AGFG1* |  | *GPRIN3* | **chr7** | *SLC37A3* |
|  | *VAV3* |  | *RALB* (or *TMEM185A*) |  | *DTHD1* |  | *DKFZp761N0624* |
|  | *GBP2* |  |  |  | *ARHGAP10* |  | *UPP1* |
|  | *CD53* |  | *TMSB10* |  | *ZFYVE28* |  | *AVL9* |
|  | *MAN1C1* |  | *TRABD2A* |  |  |  | *AK095549* (or *CR618431*) |
|  | *PIGV* |  |  | **chr5** | *CDC42SE2* |  |  |
|  | *KIAA0040* | **chr3** | *CISH* |  | *SLC22A5* |  | *SNX10* |
|  | *CAPZB* |  | *EGOT* (or *ITPR1*) |  | *MTX3* |  | *C7orf23* |
|  | *RASSF5* |  |  |  | *ADAM19* |  | *IMMP2L* (or *LRRN3*) |
|  | *PTP4A2* |  | *TRAT1* |  | *LCP2* |  |  |
|  | *PDE4DIP* (or *NBPF14* or *NBPF8*) |  | *PCYT1A* |  | *SV2C* |  | *LRRC61* |
|  |  |  | *TRAT1* |  | *MSH3* |  | *ZC3HAV1* |
|  |  |  | *C3orf18* (or *DKFZp586G2119*) |  | *KCTD16* |  |  |
|  | *MACF1* |  |  |  | *SPEF2* | **chr8** | *MYBL1* |
|  | *RABGAP1L* |  |  |  | *CD14* (or *TMCO6*) |  | *SH2D4A* |
|  | *SKI* |  | *HEMK1* |  |  |  | *CSGALNACT1* |
|  | *PTPRC* |  | *GPR171* (or *MED12L*) |  | *ERAP2* |  | *SNTB1* |
|  | *SMAP2* |  |  |  | *RNF145* |  | *MYC* |
|  | *PDE4DIP* |  | *LPP* |  | *STK10* |  | *ANKRD46* |
|  | *LYST* |  | *LRIG1* |  | *S100Z* |  | *GSDMD (or ZC3H3)* |
|  |  |  | *MUC4* |  | *GZMK* |  | *GSDMD* (or *ZC3H3*)  *TMEM71* |
| **chr2** | *UGP2* |  | *DLEC1* (or *PLCD1*) |  |  |  |  |
|  | *IL1R1 (orIL1RL2)* |  |  | **chr6** | *CCND3* |  | *MFHAS1* |
|  |  |  | *TBC1D5* |  | *CDKAL1* |  | *AP3M2* |
|  | *DPP4* |  | *HHLA2* |  | *BC030116* |  | *MYOM2* |
|  | *ARL4C* |  | *ZPLD1* |  | *ATG5* |  | *NSMCE2* |
|  | *AK001558* |  | *MB21D2* |  | *LPAL2* (or *SLC22A3*) |  | *LEPROTL1* (or *TMEM66*) |
|  | *ARHGAP15* |  | *KAT2B* |  |  |  |  |
|  | *BRE* |  | *BCL6* |  | *SGK1* |  | *BX648371* |
|  | *HECW2* |  | *TMF1* |  | *GTF2H5* |  |  |
|  | *CDKL4* |  | *RAB7A* |  | *SOGA3* |  |  |
|  | *STK17B* |  | *IFT80* (or *SMC4*) |  | *FAM65B* |  |  |
|  | *NEB* |  |  |  | *PRDM1* |  |  |
|  | *HDAC4* |  |  |  | *IRF4* |  |  |
| **chr9** | *CDKN2BAS* | chr12 | *LTA4H* | chr17 | *AXIN2* | chr20 | *ISM1* |
|  | *FGD3* |  | *SELPLG* |  | *KIAA1618* |  | *NDRG3* |
|  | *SUSD3* |  | *USP15* |  | *SLC26A11* |  |  |
|  | *CD274* |  | *HELB* |  | *PIK3R5* | **chr21** | *SAMSN1* |
|  | *TMEM2* |  | *CD69* |  | *SENP3* (or*TNSF12*) |  | *TIAM1* |
|  | *ERCC6L2* |  | *PARP11* |  |  |  | *SLC37A1* |
|  | *C9orf156* |  | *CRYL1* |  | *TMEM49* |  |  |
|  | *FNBP1* |  |  |  | *PITPNC1* | **chr22** | *GRAP2* |
|  | *MAK10* | **chr13** | *LPAR6* (or *RB1*) |  | *CYTH1* |  | *MYH9* |
|  | *GNAQ* |  |  |  | *SOCS3* |  | *HORMAD2* |
|  | *KLF9* |  | *WDFY2* |  | *FAM117A* |  |  |
|  |  |  | *RCBTB2* |  | *PER1* | **chrX** | *XPNPEP2* |
| **chr10** | *FAM45A* |  | *SCARNA13* |  | *IKZF3* |  | *ELF4* |
|  | *DUSP5* |  |  |  | *MPRIP* |  | *SUV39H1* |
|  | *BC037918* | **chr14** | *SYNE2* |  | *MMD* |  |  |
|  | *OBFC1* |  | *KIAA0247* |  |  | **STAT5A specifically detected genes**  **STAT5A specific regulated genes**  **chr1** | |
|  | *REEP3* |  | *YLPM1* | **chr18** | *SMCHD1* |  |  |
|  | *CUBN* |  | *WARS* (or *WDR25*) |  | *MBP* | **chr1** | *GBP5* |
|  | *SFMBT2* |  |  |  | *C18orf1* |  | *PRG4* |
|  | *AY007155* |  | *RAD51L1* |  | *YES1* |  | *CITED4* |
|  | *PCGF5* |  | *C14orf182* |  | *PTPN2* |  |  |
|  | *DNAJC12* |  |  |  | *MALT1* | **chr2** | *PARD3B* |
|  | *ADD3* | **chr15** | *LINS1* (or *ASB7*) |  | *LAMA3* |  | *OBFC2A* |
|  | *C10orf55* |  |  |  | *MYL12B* |  | *ST3GAL5* |
|  | *CDH23* |  | *RNF111* |  | *DLGAP1* |  | *YIPF4* |
|  | *ALOX5* |  | *MYO5A* |  | *CD226* |  | *PLCL1* |
|  | *EGR2* |  | *LIPC* |  | *LMAN1* |  | *TMEM194B* |
|  | *FAM107B* |  | *SNUPN* |  | *MYOM1* |  |  |
|  |  |  | *RORA* |  |  | **chr3** | *FRMD4B* |
| **chr11** | *IL10RA* |  | *PATL2* | **chr19** | *VRK3* (or *ZNF473*) |  | *XIRP1* |
|  | *MICAL2* |  | *AEN* |  |  |  |  |
|  | *BC038205* |  | *TRIM69* |  | *DNM2* | **chr4** | *NFKB1* |
|  | *ETS1* |  |  |  | *RYR1* |  |  |
|  | *ASAM* (or *HSPA8*) | **chr16** | *CLEC16A* |  | *CD97* | **chr5** | *IL7R* |
|  |  |  | *ZNF267* |  | *BLOC1S3* (or *TRAPPC6A*) |  |  |
|  | *NEAT1* |  | *AK307158* |  |  | **chr6** | *C6orf105* |
|  | *TRIM22* (or *TRIM5*) |  | *N4BP1* |  | *KIAA0355* |  | *TREML2* |
|  |  |  | *XYLT1* |  | *GNA15* |  | *ADTRP* |
|  | *ABCD2* (or *C12orf40*) |  | *C16orf62* |  | *ACTL9* |  | *ESR1* |
|  |  |  | *IL32* |  | *TPM4* |  | *MAP3K5* |
|  |  |  | *IL4R* |  | *B9D2* (or *CYP2F1* or *TMEM91*) |  | *LY86* |
| **chr12** | *C12orf4* |  | *CYLD* |  |  |  | *LTB* |
|  | *DDX23* |  | *ABCC1* |  |  |  |  |
|  | *PTPRO* |  |  |  |  | **chr7** | *CDK6* |
|  | *CLEC2D* | **chr17** | *RNF157* | **chr20** | *NCOA3* |  | *PHTF2* (or *TMEM60*)  *PHTF2 (or TMEM60)*  *MAD1L1* |
|  | *BC053951* |  | *SLFN12L* |  | *COMMD7* |  |  |
|  | *TPCN1* |  | *MSI2* |  | *NFATC2* |  | *MAD1L1* |
| **chr8** | *WHSC1L1* | **chr6** | *SAMD3* | chr1 | *GBP1* | chr5 | *DNAJC18* |
|  | *ST3GAL1* |  |  |  | *DNM3* |  | *MCC* |
|  |  | **chr8** | *NDRG1* |  | *TP53BP2* |  | *HAVCR1* |
| **chr9** | *TNFSF8* |  | *DENND3* |  | *LMO4* |  | *CD180* |
|  |  |  | *ANK1* |  | *LRRC8B* |  | *REEP5* (or *ZRSR2*)  *REEP5 (or ZRSR2)*  *C5orf39 (or LOC153684)* |
| **chr10** | *CUGBP2* |  | *TOX* |  | *LY9* |  |  |
|  | *SEC31B* |  |  |  | *ASH1L* |  | *C5orf39* (or *LOC153684*)  *ANKRD55*  *C5orf39 (or LOC153684)*  *ANKRD55* |
|  |  | **chr9** | *SUSD1* |  | *HFE2* |  |  |
| **chr11** | *DENND5A* |  | *RALGDS* |  | *KIAA0319L* |  |  |
|  |  |  |  |  |  |  |  |
| **ch12** | *CCDC53* | **chr10** | *CAMK1D* | **chr2** | *B3GNT2* | **chr6** | *SNX9* |
|  | *RHOF* (or *SETD1B*) |  | *APBB1IP* |  | *DOCK10* |  | *FILIP1* |
|  |  |  |  |  | *XRCC5* |  | *PTPRK* |
|  | *SH2B3* | **chr11** | *IFITM1* |  | *NFE2L2* |  | *TIAM2* |
|  |  |  | *BIRC3* |  | *RBMS1* |  | *FKBP5* |
| **chr13** | *A2LD1* |  |  |  | *NOL10* |  | *IL22RA2* |
|  |  | **chr14** | *C14orf182* |  | *KLRAQ1* |  | *IRF4* |
| **chr14** | *SAMD4A* |  | *PPP2R5C* |  | *MGAT4A* |  | *HIVEP1* |
|  |  |  |  |  | *KCMF1* |  | *HSPA1A* |
| **chr17** | *SSH2* | **chr16** | *METTL9* |  | *TGFA* |  | *THEMIS* |
|  | *TOM1L2* |  | *PRMT7* |  |  |  | *SUMO4* |
|  | *SMG6* |  | *TK2* | **chr3** | *TGFBR2* |  | *C6orf89* |
|  |  |  |  |  | *CD47* |  |  |
| **chr18** | *SETBP1* | **chr18** | *NPC1* |  | *UBE2E2* | **chr7** | *HDAC9* |
|  |  |  |  |  | *IGSF11* (or *RGS20*) |  | *PRKAR2B* |
| **chr19** | *GNG7* | **chr19** | *CNTD2* |  |  |  | *GIMAP5* |
|  |  |  | *FAM83E* |  | *SNRK* |  | *SSH2* |
| **chr20** | *STX16* |  | *RPL18* (or *SPHK2*) |  | *MBNL1* |  | *GSTK1* |
|  |  |  |  |  |  |  | *AUTS2* |
| **chr22** | *TBC1D10A* |  | *FLT3LG* | **chr4** | *ANTXR2* |  | *SLC26A4* |
|  | *ST3GAL5* |  |  |  | *TNIP3* |  | *VOPP1* |
|  |  | **chr20** | *PREX1* |  | *G3BP2* |  | *GIMAP7* |
| **STAT5A dominantly detected genes** | |  | *ZNF217* |  | *TMEM156* |  | *ELMO1* |
|  |  |  |  |  | *MND1* |  |  |
| **chr1** | *DNAJC6* | **chr21** | *CBS* |  | *CNOT6L* | **chr8** | *RNF19A* |
|  | *S100A11* |  |  |  | *FRYL* |  | *KIAA0146* |
|  | *LRRC8C* | **STAT5B specifically detected genes** | |  | *STIM2* |  | *EIF2C2* |
|  | *FMOD* |  |  |  | *FBXW7* |  | *NCALD* |
|  | *CHRM3* | **chr1** | *MCOLN3* |  | *KIAA0914* |  | *ZNF250* |
|  |  |  | *TRAF3IP3* |  |  |  | *ATP6V1H* |
| **chr2** | *CFLAR* |  | *ZNF238* | **chr5** | *LNPEP* |  | *TPD52* |
|  | *STAT1* |  | *DSTYK* |  | *TNFAIP8* |  | *LAPTM4B* |
|  |  |  | *ARNT* |  | *UBTD2* |  | *MTSS1* |
| **chr3** | *MAPKAPK3* |  | *SWT1* |  | *TRIO* |  | *TG* |
|  |  |  | *RYR2* |  | *NDFIP1* |  | *PAG1* |
| **chr5** | *PPP2R2B* |  | *LPGAT1* |  | *MCC* |  | *LETM2* |
|  | *CSNK1G3* |  | *IFI16* |  | *TBCA* |  |  |
| **chr9** | *DOCK8* | chr13 | *KLF12* | **chr21** | *CASS4* | **chr2** | *DGKD* |
|  | *GCNT1* |  |  |  | *TGIF2* |  | *DNAH6* |
|  | *OSTF1* | **chr14** | *PTGER2* |  | *STK4* |  | *TRAK2* |
|  | *SEMA4D* |  | *CCDC88C* |  | *TOMM34* |  | *SOS1* |
|  | *GPR107* |  | *NIN* |  | *IDH3B* |  | *ANKRD44* |
|  | *TNSF8* |  | *HIF1A* |  |  |  | *SLC4A5* |
|  | *TRAF1* |  | *GLRX5* | **chr22** | *ZNRF3* |  | *PTPN4* |
|  | *SPTLC1* |  | *SPTLC2* |  | *abParts* |  | *NCK2* |
|  |  |  | *C14orf19* |  | *RAC2* |  | *ACOXL* |
| **chr10** | *TM9SF3* |  |  |  | *APOL6* |  | *RBKS* |
|  | *PDCD4* | **chr15** | *CRTC3* |  | *ENTHD1* |  |  |
|  | *ANK3* |  | *SLCO3A1* |  |  | **chr3** | *ST6GAL1* |
|  | *DIP2C* |  | *MAP2K1* | **chrX** | *OPHN1* |  | *TNIK* |
|  | *PIP4K2A* |  | *BCL2A1* |  | *TMSB4X* |  | *CBLB* |
|  | *RBM17* |  |  |  |  |  | *TNFSF10* |
|  | *FRMD4A* | **chr16** | *RM12* | **STAT5B dominantly detected genes** | |  | *OXNAD1* |
|  | *RAB11FIP2* |  | *CMTM3* |  |  |  | *FHIT* |
|  | *IDI1* |  | *WWOX* | **chr1** | *VPS13D* |  | *ARHGEF3* |
|  |  |  | *ZNF768* |  | *CD48* |  | *FNDC3B* |
| **chr11** | *GAB2* |  | *CRAMP1L* |  | *DPH5* |  | *UBA3* |
|  | *STIP1* |  | *GLG1* |  | *GREM2* |  | *FOXP1* |
|  | *EED* |  |  |  | *RCAN3* |  | *NFKBIZ* |
|  | *TMEM123* | **chr17** | *SKAP1* |  | *CD55* |  | *COLQ* |
|  | *C11orf31* |  | *EIF1* |  | *NBPF1* |  | *ATXN7* |
|  | *B3GNT6* |  | *TNRC6C* |  | *CD247* |  |  |
|  | *CD5* |  | *RFT1* |  | *TARBP1* | **chr4** | *TLR6* |
|  | *AMBRA1* |  | *MEOX1* |  | *TNFRSF1B* |  | *INPP4B* |
|  | *CARS* |  | *COPZ2* |  | *RAP1A* |  | *CLNK* |
|  | *FRMD8* |  | *C17orf97* |  | *RSBN1* |  | *ARSJ* |
|  | *IGSF9B* |  | *HEXDC* |  | *LPAR3* |  | *COL25A1* |
|  | *FCHSD2* |  |  |  | *ADAMTSL4* (or *MCL1*) |  | *AFF1* |
|  | *CD6* | **chr18** | *MIB1* |  |  |  | *SCLT1* (or *C4orf33*)  *SCLT1 (or C4orf33)* |
|  |  |  | *C18orf25* |  | *SLC9A11* |  |  |
| **chr12** | *EP400NL* |  | *TAF4B* |  | *AIM2* |  |  |
|  | *KDM2B* |  | *RNF125* |  | *PMF1* | **chr5** | *ITK* |
|  | *TUWD12* |  |  |  | *FAM102B* |  | *PPAP2A* |
|  | *CCDC64* | **chr19** | *ZC3H4* |  | *TNFRSF9* |  | *LOC257358* |
|  | *ETV6* |  | *CEACAM1* |  | *CYCSP52* (*or ETV3*) |  | *TNFAIP8* |
|  | *NUP107* |  | *SIGLEC8* |  |  |  | *FAM71B* |
|  | *ARHGDIB* |  | *MKNK2* |  |  |  | *HRH2* |
|  |  |  | *SAE1* | **chr2** | *WIPF1* |  | *GNPDA1* |
| **chr13** | *TNFSF13B* |  | *ZNF101* |  | *PGAP1* |  |  |
|  | *USP12* |  |  |  | *CYTIP* | **chr6** | *EXOC2* |
|  | *SACS* | **chr20** | *DIDO1* |  | *IMMT* |  | *NEDD9* |
|  | *TBC1D4* |  | *SAMHD1* |  | *CASP8* |  | *ATXN1* |
|  | *UBAC2* |  | *ZBP1* |  | *KLF7* |  | *HIVEP2* |
|  | *AK056689* |  | *GTSF1L* |  | *IL18RAP* |  | *PIM1* |
| chr6 | *HBS1L* | **chr13** | *LCP1* |  |  |  |  |
|  | *BACH2* |  | *CYSLTR2* |  |  |  |  |
|  | *PPP1R18* |  |  |  |  |  |  |
|  | *DHX16* | **chr14** | *ARHGAP5* |  |  |  |  |
|  |  |  | *PRKCH* |  |  |  |  |
| **chr7** | *FLJ43663* |  | *RGS6* |  |  |  |  |
|  | *WASL* |  | *RPS6KA5* |  |  |  |  |
|  | *OSBPL3* |  | *GNG2* |  |  |  |  |
|  | *MDFIC* |  |  |  |  |  |  |
|  | *MDFIC* | **chr15** | *RAB11A* |  |  |  |  |
|  | *IKZF1* |  | *MEGF11* |  |  |  |  |
|  | *ATXN7L1* |  | *BC036442* |  |  |  |  |
|  |  |  |  |  |  |  |  |
| **chr8** | *RAB11FIP1* | **chr16** | *CLUAP1*  *(orC16orf90*) |  |  |  |  |
|  |  |  |  |  |  |  |  |
| **chr9** | *UGCG* |  | *CES8* |  |  |  |  |
|  | *ARPC5L* (*or RPL35*) |  |  |  |  |  |  |
|  |  | **chr17** | *RPA1* |  |  |  |  |
|  | *MLLT3* |  | *ERN1* |  |  |  |  |
|  | *KDM4C* |  | *C17orf75* |  |  |  |  |
|  | *SMARCA2* |  | *PRKCA* |  |  |  |  |
|  | *PCSK5* |  |  |  |  |  |  |
|  | *TRIM14* | **chr18** | *BCL2* |  |  |  |  |
|  |  |  | *SMCHD1* |  |  |  |  |
| **chr10** | *ARID5B* |  | *TTC39C* |  |  |  |  |
|  | *CREM* |  | *LPIN2* |  |  |  |  |
|  | *PIP4K2A* |  |  |  |  |  |  |
|  | *KLF6* | **chr19** | *CYP2F1* |  |  |  |  |
|  | *RTKN2* |  | *EPS15L1* |  |  |  |  |
|  | *IL2RA* |  | *ACTN4* |  |  |  |  |
|  | *ADK*  *IFIT3 (or LIPA)* |  | *PBX4* |  |  |  |  |
|  | *IFIT3* (*or LIPA*) |  | *NUCB1 (or TULP2)* |  |  |  |  |
|  |  |  |  |  |  |  |  |
| **chr11** | *CASP12* |  |  |  |  |  |  |
|  | *PSMA1* | **chr20** | *PTPN1* |  |  |  |  |
|  | *CASP4* |  | *ZNF831* |  |  |  |  |
|  | *TMEM123* |  |  |  |  |  |  |
|  | *PDE3B* | **chr21** | *IFNGR2* |  |  |  |  |
|  | *SPTY2D1* |  | *TMEM50B* |  |  |  |  |
|  |  |  | *RUNX1* |  |  |  |  |
| **chr12** | *FLJ40142* |  |  |  |  |  |  |
|  | *CRADD* | **chr22** | *TNRC6B* |  |  |  |  |
|  | *FGFR1OP2* |  | *IL2RB* |  |  |  |  |
|  | *FAM113B* |  |  |  |  |  |  |
|  |  |  |  |  |  |  |  |
|  |  |  |  |  |  |  |  |

**Supplementary Figure legend**

**Supplementary Fig. 1 Validation of anti-STAT5A Ab and anti-STAT5B Ab by Western blot**

**Suppl Fig. 1** proves the specificities of anti-STAT5A Ab and anti-STAT5B Ab for each protein by western blot technique. Cytoplasmic cell lysate from CD4+ T cells was used for each analysis. Protein was detected with a 1:1000 dilution of either anti-STAT5A Ab and/or anti-STAT5B Ab.
